# Decline of Neuropsychological Abilities in a Large Sample of Patients with Multiple Sclerosis: A Two-Year Longitudinal Study

**DOI:** 10.3389/fnhum.2016.00282

**Published:** 2016-06-08

**Authors:** Martina Borghi, Sara Carletto, Luca Ostacoli, Francesco Scavelli, Lorenzo Pia, Marco Pagani, Antonio Bertolotto, Simona Malucchi, Alessio Signori, Marco Cavallo

**Affiliations:** ^1^Clinical Psychology and Psychosomatics Service, University Hospital San Luigi Gonzaga, University of TurinOrbassano, Italy; ^2^SAMBA (SpAtial, Motor and Bodily Awareness) Research Group, Department of Psychology, University of TurinTurin, Italy; ^3^Neuroscience Institute of Turin (NIT), University of TurinTurin, Italy; ^4^Institute of Cognitive Sciences and Technologies, Consiglio Nazionale delle Ricerche (CNR), RomeItaly; ^5^Neurologia 2 – CRESM (Regional Reference Centre for Multiple Sclerosis), “San Luigi Gonzaga” Hospital Medical SchoolOrbassano, Italy; ^6^Department of Health Sciences (DISSAL), Section of Biostatistics, University of GenoaGenoa, Italy; ^7^Faculty of Psychology, eCampus UniversityNovedrate, Italy; ^8^Department of Mental Health, Azienda Sanitaria Locale Torino 3Collegno, Italy

**Keywords:** cognitive deficits, linear mixed models, longitudinal analysis, multiple sclerosis, neuropsychology

## Abstract

**Objective**: In this longitudinal study, we monitored two large groups of multiple sclerosis (MS) patients and healthy controls (HC) for 2 years, with the aim of comparing their neuropsychological profile over time.

**Method**: Three hundred and twenty-two patients with MS and 303 HC were administered the Brief Repeatable Battery of Neuropsychological tests (BRB-N); neuropsychiatric measures were also administered. Two follow-ups were scheduled at 1 and 2 years, respectively.

**Results**: A linear mixed model (LMM) with random intercept was run by considering participants’ performance on each test of the BRB-N at the three assessment points (baseline and follow-ups) as the within-subjects variable, and group (patients and controls) as the between-subjects factor. The interaction term was statistically significant for the tests: Symbol Digit Modalities test (SDMT) (*p* = 0.044), Paced Auditory Serial Addition test (PASAT) (*p* = 0.011) and Word List Generation (WLG) (*p* < 0.001), whereas for the PASAT-3 approached statistical significance (*p* = 0.05). In addition, a LMM with random intercept was also run by identifying three groups (controls, relapsing-remitting course of MS (i.e. RR-MS), and prog-MS). The interaction term was statistically significant for: PASAT-3 (*p* = 0.017), PASAT-2 (*p* = 0.0026), and WLG (*p* = 0.0022).

**Conclusions**: Our results corroborate on a very large scale evidence that the abilities tapped by the tasks SDMT, PASAT and WLG are particularly sensitive to MS, and further extend this issue by showing that these abilities are likely to be more sensitive than others to the progression of the disease, as compared to HC.

## Introduction

Neurological and neuropsychiatric conditions are often characterized by significant deficits that reduce significantly patients’ abilities and their overall quality of life (Borghi et al., 2013; Cavallo et al., 2013a,b). Multiple Sclerosis (MS) is a chronic neurodegenerative condition characterized by white matter lesions, axonal damage, and cerebral atrophy (Kornek and Lassmann, 2003; Bermel and Bakshy, 2006) and frequently associated with psychological problems (Ostacoli et al., 2013). Cognitive deficit is a frequent clinical feature of MS, ranging from 30% to 70% of patients (Rao et al., 1991; Borghi et al., 2013). MS negatively affects various cognitive abilities such as speed of processing, attention, new learning, executive functions, and memory (DeLuca et al., 2004; Bodling et al., 2012; Borghi et al., 2013). Cognitive deficits do not imply only problems in performing accurately neuropsychological tests, but it is typically related to patient’s reduced functional status, and has a serious impact on patients’ quality of life (Kalmar et al., 2008; Fernández et al., 2011).

An important issue pertains to the possible decline of patients’ neuropsychological abilities over time, as compared to healthy controls (HC). In the last decade, some studies have started to tackle this substantial issue. In one of the first studies, Piras et al. (2003) recruited 12 patients affected by relapsing-remitting MS, to monitor disease evolution, cognitive dysfunction and cerebral lesion burden over 8.5 years. Their results showed the presence of mild cognitive impairment in MS, mainly related to a diminished information processing ability over time. In addition, magnetic resonance imaging (MRI) lesions did not correlate with the decline of cognitive abilities. A few years later, Camp et al. (2005) performed a multi-center European study involving an overall size of 99 patients affected by primary progressive MS, with the aim of investigating cognitive and MRI changes over 2 years. Their results did not show the presence of a significant decline of neuropsychological abilities over time, and only a very weak relation between neuropsychological and structural neuroimaging measures was found. Huijbregts et al. (2006) included in their study 30 secondary-progressive MS patients, 25 primary-progressive MS patients, and 33 controls and investigated a 2-year follow-up of neuropsychological performance on the Brief Repeatable Battery of Neuropsychological tests (Rao et al., 1990). Their results indicated that both progressive MS did not show a learning effect on the Symbol Digit Modalities Test (SDMT) and the Paced Auditory Serial Addition Test (PASAT) as compared to controls, indicating that performance on tasks requiring multiple abilities such as visuo-spatial ability and processing speed (for SDMT) or working memory and processing speed (for PASAT), is most likely to decline over time.

More recently, Denney et al. (2008) recruited 24 MS patients and 25 HC and monitored their neuropsychological profile over a 3-year period. They showed that patients’ performance on measures of speed of processing declined significantly over time, as compared to the control group, whereas other domains such as problem-solving abilities or verbal memory remained relatively stable during the course of the study. A similar pattern of results was recently shown by Hankomäki et al. (2014). In another recent study, Loitfelder et al. (2014) recruited 13 MS patients and 15 HC who underwent MRI including functional magnetic resonance imaging (fMRI) and neuropsychological exams at baseline and at follow-up (median: 20 months): from a neuropsychological point of view, their results showed that cognitive abilities remained largely stable in the patients over time. Very recently, Nygaard et al. (2015) investigated neuropsychological progression over 1 year in relapsing-remitting MS patients (*N* = 73) and matched HC (*n* = 61). Patients were divided into those showing “evidence of disease activity” or showing “no evidence of disease activity”. While the two groups had similar disability levels at baseline, they differed in their level of disability at follow-up. Regarding the domain of cognition, both patients group were stable. Lastly, Van Schependom et al. (2015) collected neuropsychological data on *N* = 514 MS patients using the Neuropsychological Screening Battery for MS and the SDMT. Information processing speed was the domain most widely affected in MS patients, and also the first cognitive ability to be compromised in MS.

At this point in time, no studies have investigated changes over time in neuropsychological abilities in a very large group of MS patients, as compared to a very large group of HC. In addition, little is known about the evolution of the neuropsychological profile in progressive MS patients, as compared to relapsing-remitting MS patients, and HC. Thus, in the present prospective longitudinal study we monitored two very large groups of MS patients (*N* = 322) and HC (*N* = 303) for 2 years, with two aims: (1) investigating the changes in neuropsychological abilities in MS patients as compared to HC to differentiate what is normal decline over time and what is MS related decline; and (2) detecting possible differences over time in the neuropsychological profile of different subgroups of patients affected by different courses of the disease (relapsing-remitting vs. progressive course of MS).

## Materials and Methods

A detailed presentation of the methods of this study has been already reported in an our previous article aimed at investigating the determinants of cognitive impairment in MS (Borghi et al., 2013).

### Participants

Three hundred and twenty-two patients with MS (97 males and 225 females) were consecutively recruited from May 2010 to June 2012 from the CReSM (Regional Reference Center for Multiple Sclerosis, affiliated with the University Hospital “San Luigi Gonzaga” of Orbassano, Italy), an Italian reference center for the diagnosis and treatment of patients with MS. All of the patients underwent detailed biological and clinical investigations, and received a diagnosis of MS, according to the standard International criteria (Polman et al., 2011), by neurologists expert in the diagnosis of MS. Patients with possible MS or clinically isolated syndrome were not included in the study. More precisely, 89% of the patients suffered from a relapsing-remitting course of the disease, while the others were characterized by a progressive course of the condition.

Inclusion criteria were as follows: definite diagnosis of MS according to the standard International criteria (Polman et al., 2011); more than 18 years old; fluent Italian speakers. Patients under high dosage of corticosteroids at the time of the beginning of the study were temporarily excluded, and they were administered the neuropsychological battery 1 month after the interruption of the drug treatment. At the time of enrolment, 53% of patients were under immunomodulatory therapy; 12% of patients under immunosuppressive therapy; 18% of patients under monoclonal antibodies therapy; and 17% of patients were not taking any medication. Exclusion criteria were as follows: presence of severe psychiatric disorders such as psychosis or bipolar disorder; presence of severe medical conditions other than MS such as diabetes, stroke or traumatic brain injury; drug or alcohol abuse; suicide attempts; overt dementia; and serious eye disorders (such as diplopia).

Three hundred and three HC (93 males and 210 females) were recruited among the health professionals working at the same hospital and among caregivers of patients admitted to different hospital departments. None of the controls were consanguineous of MS patients included in the present research project. A clinical interview based on Green (2000) allowed us to rule out in our control group the presence of significant neurological or psychiatric disorders, of alcohol and drug abuse, or of other relevant clinical conditions. Informed written consent was obtained from all of the participants, in accordance with the Declaration of Helsinki. This study was granted approval by the Research Ethics Committee of the “San Luigi Gonzaga” Hospital Medical School of Orbassano, Turin (Italy).

### Procedure

The participants were administered the neuropsychiatric and neuropsychological measures detailed below at the University Hospital “San Luigi Gonzaga” of Orbassano (Italy) by expert clinicians (MB, SC, FS).

#### Neuropsychiatric Measures

The participants were administered the following two measures: *the Hospital Anxiety and Depression Scale* (HADS; Zigmond and Snaith, 1983), a 14-item self-assessment scale that provides a valid and reliable measure of severity of anxiety and depression; and *the Fatigue Severity Scale* (FSS; Krupp et al., 1989), a 9-item one-dimensional questionnaire assessing the severity of fatigue. Lastly, patients with MS received a score from their neurologists on the *Expanded Disability Status Scale* (EDSS; Kurtzke, 1983), to monitor their level of disability presented at the time of the current neuropsychological assessment.

#### Neuropsychological Measures

All of the participants were administered the Brief Repeatable Battery of Neuropsychological Tests (BRB-N) for Multiple Sclerosis (Rao et al., 1990), a neuropsychological battery sensitive to the cognitive deficits that typically characterize MS. The BRB-N encompasses the following tests: *the Selective Reminding Test (SRT)*, a test for verbal memory that provides measurement of learning and delayed recall capacity. It yields three different scores: the SRT-Long Term Storage (SRT-LTS), that provides a measure of the storage capacity in long-term memory; the SRT-Consistent Long Term Retrieval (SRT-CLTR), that provides a measure of the consistency of the recovery in long-term memory; the SRT-Delayed (SRT-D) a delayed recall of the words of the previously learned. *SPART*, a test of learning and delayed recall of visuo-spatial items. It yields two scores: the SPART immediate recall score, and the SPART delayed recall score. *SDMT*, a test of attention and of speed of information processing. *PASAT*, that assesses the speed of information processing, the working memory, and the sustained attention. It encompasses two separate sub-tests (PASAT-2 and PASAT-3) in which the interval between two consecutive items changes (2 or 3 s, respectively). Finally, *Word List Generation (WLG)*, a semantic verbal fluency task. For all of the BRB-N tasks, higher scores mean better performance. Furthermore, an additional cognitive measure was administered to all of the participants at the beginning of the study: *the Brief Intelligence Test* (Test di Intelligenza Breve, or TIB; Colombo et al., 2002), functionally equivalent to the National Adult Reading Test (Nelson, 1982), a well-established measure aiming at estimating the pre-morbid Intelligence Quotient.

Each participant was administered the BRB-N three times: at the beginning of the study (T_0_), after 1 year (T_1_), and after 2 years (T_2_). In order to minimize practice effects, two parallel versions of the BRB-N were used: half of the participants started with version A, then were administered version B at the 1-year follow-up, and lastly were administered version A again at the 2-year follow-up (i.e., A-B-A), whereas half of the participants did the other way around (i.e., B-A-B). In doing so, the same version of the neuropsychological battery was repeated by each participant only after 2 years.

As in our previous study (Borghi et al., 2013), to define the construct of “cognitive impairment”, we used the criteria proposed by Amato et al. (2010), a failure in at least two BRB-N tests, with scores at least 1.5 SD below the scores of HC. Thus, if a patient had zero or one BRB-N test score at least 1.5 SD below that of HC the patient was considered to have no cognitive impairment. If a patient had two or more BRB-N test scores at least 1.5 SD below that of HC, the patient was considered to have cognitive impairment. In order to differentiate the degree of severity of deficits, if a patient had two BRB-N test scores below that of HC, the patient was considered to have a *mild* degree of cognitive impairment. If a patient had three BRB-N test scores below that of HC, the patient was considered to have *moderate* cognitive impairment. Finally, if a patient had four or more BRB-N test scores below that of HC, the patient was considered to have *severe* cognitive impairment.

### Statistical Analyses

Statistical analyses were performed using Stata (StataCorp.) version 13. Parametric tests were used due to the large sample sizes and because graphical exploration of the data indicated an acceptable distribution of the variables of interest. Statistical analyses were as follows: firstly, the comparison of the two groups (patients and controls) was performed via a series of *t*-tests for independent samples by considering demographic and clinical variables: *p*-values were adjusted for multiple comparisons using the false-discovery rate (FDR) approach. Secondly, we investigated the presence and the degree of cognitive impairment in our sample of patients with MS at the three assessment points (e.g., T_0_, T_1_ and T_2_). Thirdly, in order to investigate the longitudinal effect on neuropsychological performances, univariate and multivariate linear mixed models (LMM) with random intercept (function Mixed in Stata, v.13) were performed for each scale investigated. Particularly, the interaction term “group × time” was introduced into the model to test statistically whether the trajectories over time were significantly different in MS patients and healthy subjects. Furthermore, in order to investigate the longitudinal neuropsychological profile of patients with different courses of the disease, MS patients were also divided into two sub-groups, depending on their disease course (RR-MS, and prog-MS, respectively). LMMs were performed in the same manner to investigate differences across these sub-groups. In this last analysis, *p*-values were adjusted for multiple comparisons using the FDR control approach. To correct the univariate trajectories for possible confounders, patients’ socio-demographic variables (e.g., age, gender, education, job status) found to be significantly associated with the single neuropsychological scales were introduced in a final multivariable model. Furthermore, the addition of a random slope into the LMMs was also considered by comparing model goodness of fit with and without the random slope entered based on Akaike Information Criterion (AIC) and Bayesian Information Criterion (BIC). A *p*-value lower than 0.05 was considered statistically significant.

## Results

At the baseline, the two groups of participants were well matched for gender, pre-morbid IQ and years of formal education, whereas a difference in age was detected, with patients being younger than HC.

### Neuropsychiatric Measures

Five patients and three HC refused to complete the HADS. One patient and five HC did not complete the FSS. All of the other scores have been collected and used in the present analyses. The two groups of participants differed in HADS-anxiety (*t*_(615)_ = 2.522, *p* < 0.05) and HADS-depression (*t*_(615)_ = 4.740, *p* < 0.01). In addition, they also differed in the level of fatigue (*t*_(574)_ = 10.654, *p* < 0.01), in keeping with previously published studies. Table 1 reports the demographic and neuropsychiatric variables of interest.

**Table 1:** **Demographic and clinical variables of patients with multiple sclerosis (MS) and healthy controls (HCs) at baseline**.

Variable	MS patients mean (SD) (*n* = 322)	Healthy controls mean (SD) (*n* = 303)	*t*-test (df) *or* χ^2^	*p*-value*
**Participants’ characteristics**
Age in years	41.98 (11.37)	44.89 (11.64)	3.161 (623)	0.0032
Gender (M:F)	97.225	93.210	χ^2^ = 0.024	0.88
Education in years	12.86 (3.61)	13.18 (3.94)	1.064 (623)	0.33
IQ	111.78 (6.50)	112.37 (6.53)	1.121 (623)	0.33
Duration of illness in years	9.16 (7.13)	–	–
**Clinical measures**
HADS–anxiety	6.67 (3.55)	5.96 (3.42)	2.522 (615)	0.019
HADS–depression	5.70 (3.91)	4.36 (3.11)	4.740 (615)	<0.001
HADS–total	12.37 (6.72)	10.32 (5.87)	4.047 (615)	<0.001
FSS	35.74 (15.18)	24.25 (11.52)	10.654 (617)	<0.001
EDSS	2.37 (1.90)	–	–	

*, Adjusted for multiple comparisons with false-discovery rate (FDR) approach; df, degrees of freedom; EDSS, Expanded Disability Status Scale; FSS, Fatigue Severity Scale; HADS, Hospital Anxiety and Depression Scale; IQ, Intelligence Quotient.

### Neuropsychological Measures

**Figure 1:**
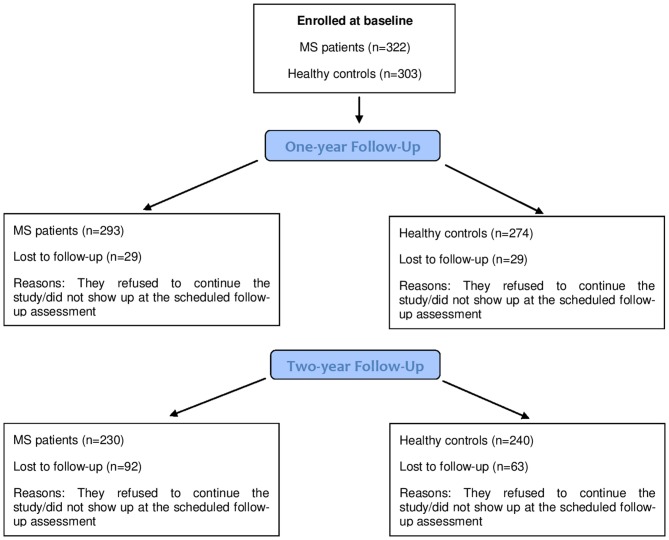
**Number of participants (Multiple Sclerosis (MS) patients and healthy controls (HCs)) at each assessment point**.

As a typical issue in longitudinal studies, not all the participants were available at the two scheduled follow-ups (1 and 2 years, respectively). Figure 1 reports a detailed indication of the participants available at each assessment point.

Firstly, we investigated the presence and the degree of cognitive impairment in our sample of patients with MS at the three assessment points (e.g., T_0_, T_1_ and T_2_). Table 2 reports the proportion of MS patients cognitively impaired at the three assessment stages, and their degree of cognitive impairment.

**Table 2:** **Proportion of MS patients cognitively impaired at the three assessment points, and their degree of cognitive impairment**.

	Cognitively impaired?		Degree of cognitive impairment		
Assessment point	NO	YES	Mild	Moderate	Severe
T0: Baseline (*N* = 322)	*N* = 213 (66.1%)	*N* = 109 (33.9%)	*N* = 35 (32.1%)	*N* = 28 (25.7%)	*N* = 46 (42.2%)
T1: After 1 year (*N* = 293)	*N* = 178 (60.8%)	*N* = 115 (39.2%)	*N* = 52 (45.2%)	*N* = 24 (20.9%)	*N* = 39 (33.9%)
T2: After 2 years (*N* = 230)	*N* = 154 (67.0%)	*N* = 76 (33.0%)	*N* = 25 (32.9%)	*N* = 19 (25.0%)	*N* = 32 (42.1%)

Secondly, we were interested in investigating the longitudinal effect on participants’ performance on the BRB-N tests. As expected, in all of the measures a significant effect of “group” was detected, as performances of patients and HC significantly differed in all of the BRB-N tests with patients getting lower scores than controls. However, for the aim of our study, we were mainly curious about the interaction between “group” and “time”: interestingly, the interaction between the two factors was statistically significant for the SDMT (*p* = 0.044; adjusted for baseline age), the PASAT-2 (*p* = 0.011; adjusted for baseline age) and the WLG (*p* < 0.001; adjusted for baseline age), and approached statistical significance for PASAT-3 (*p* = 0.05; adjusted for baseline age). Table 3 reports the details of participants’ performance at each assessment point, showing a general modest improvement at the 1-year follow-up, and a significant decay of performance between the two follow-ups on the tests just mentioned.

**Table 3:** **Neuropsychological measures in MS patients and healthy controls (HC) at each assessment point (for TIB-errors, lower scores mean better performance; for all of the other tests, higher scores mean better performance)**.

Variable	T_0_ MS HC	Delta T_1_-T_0_^a^ MS HC	Delta T_2_-T_0_^a^ MS HC	Delta T_2_-T_1_^a^ MS HC	*p*-value interaction time × group^∧^
TIB–IQ	111.78 (6.50)	–	–		
	112.37 (6.53)
TIB–errors	4.53 (5.29)	–	–		
	4.14 (5.01)
SRT–LTS	38.35 (14.72)*	3.99 (0.77)	4.65 (0.80)	0.66 (0.81)	0.96
[range 0–72]	44.34 (13.12)	4.19 (0.74)	4.51 (0.81)	0.31 (0.82)	
SRT–CLTR	28.12 (14.49)*	4.40 (0.85)	5.06 (0.89)	0.66 (0.93)	0.6
[range 0–72]	34.72 (14.63)	3.80 (0.87)	5.77 (0.88)	1.97 (0.92)	
SPART	18.48 (5.50)*	2.21 (0.30)	1.61 (0.32)	−0.60 (0.33)	0.73
[range 0–30]	19.96 (4.61)	1.90 (0.31)	1.32 (0.32)	−0.58 (0.32)	
SDMT	46.96 (13.10)*	2.21 (0.47)	0.21 (0.48)	−2.00 (0.45)	0.049
[range 0–110]	52.17 (10.42)	1.85 (0.48)	1.33 (0.47)	−0.52 (0.45)	
PASAT–3	39.70 (12.62)*	3.31 (0.53)	2.58 (0.59)	−0.73 (0.57)	0.05
[range 0–60]	41.86 (10.82)	3.03 (0.54)	4.13 (0.56)	1.10 (0.55)	
PASAT–2	27.37 (10.42)*	2.42 (0.52)	2.81 (0.61)	0.39 (0.58)	0.011
[range 0–60]	30.94 (10.54)	2.26 (0.52)	4.90 (0.57)	2.64 (0.55)	
SRT–D	7.44 (2.54)*	0.49 (0.11)	0.48 (0.12)	−0.01 (0.12)	0.43
[range 0–12]	8.29 (2.22)	0.49 (0.11)	0.68 (0.12)	0.19 (0.12)	
SPART–D	6.51 (2.30)*	0.60 (0.13)	0.46 (0.14)	−0.14 (0.14)	0.3
[range 0–10]	6.90 (2.02)	0.86 (0.13)	0.49 (0.14)	−0.37 (0.14)	
WLG	22.29 (5.70)*	2.66 (0.38)	0.53 (0.34)	−2.13 (0.41)	<0.001
	23.28 (5.50)	4.08 (0.39)	2.37 (0.33)	−1.71 (0.41)	

*a, results reported as delta coefficient (standard error); ^∧^obtained from linear mixed model; PASAT, Paced Auditory Serial Addition Test; SDMT, Symbol Digit Modalities Test; SPART, Spatial Recall Test; SPART–D, Spatial Recall Test-Delayed; SRT–CLTR, Selective Reminding Test-Consistent Long Term Retrieval; SRT–D, Selective Reminding Test-Delayed; SRT–LTS, Selective Reminding test-Long Term Storage; TIB–errors, Brief Intelligence Test-Errors; TIB–IQ, Brief Intelligence Test–Intelligence Quotient; WAIS–Voc, WAIS Vocabulary; WLG, Word List Generation*.

**Figure 2:**
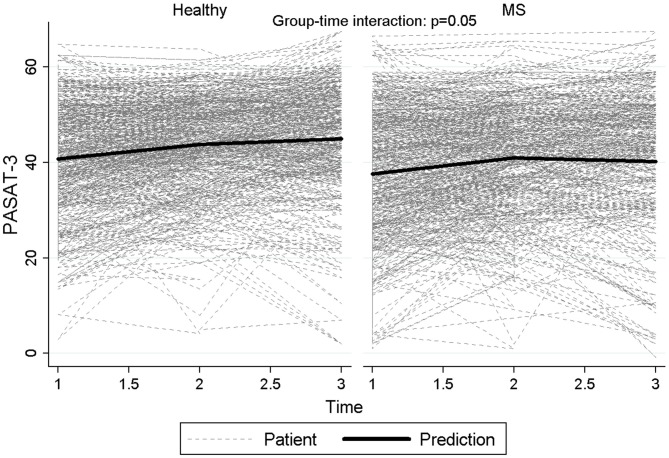
**Longitudinal performance of the two groups of participants on the PASAT-3 at the three assessment points**.

Figure 2 reports graphically the longitudinal performance of the two groups of participants on the PASAT-3 at the three assessment points.

**Figure 3:**
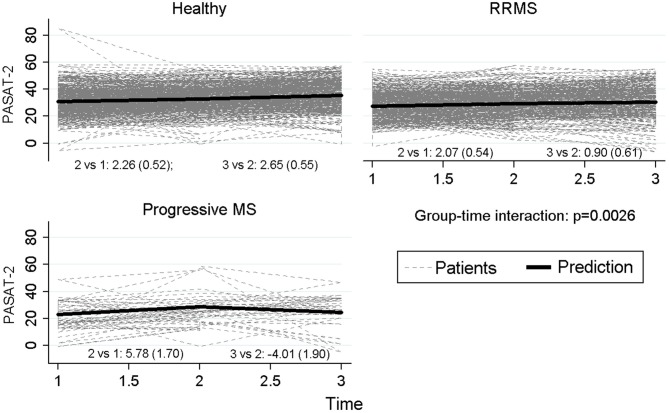
**Longitudinal performance of the three groups of participants on the PASAT-2 at the three assessment points**.

Besides, in order to investigate the longitudinal neuropsychological profile of patients with different course of the disease, we considered three groups of participants: HC, patients with a relapsing-remitting course of MS (i.e., RR-MS), and patients with a progressive course of the disease (i.e., prog-MS). Again, we were primarily curious about the interaction between “group” and “time”. The interaction between the two factors was statistically significant for PASAT-3 (*p* = 0.017), with a significant difference between HC and the progressive group of patients (*p* = 0.016), whereas the differences between the two groups of patients (*p* = 0.057) and between RRMS and HC (*p* = 0.21) did not reach statistical significance. Particularly PASAT-3 values for progressive patients at time 3 reverted to baseline values after an increase at time 2. Similar results were observed for PASAT-2 (*p* = 0.0026), with progressive MS significantly different both from HC (*p* = 0.0072) and RRMS (*p* = 0.033) and with the difference between relapsing MS and HC approaching statistical significance (*p* = 0.05). Also for the score of WLG, a significant difference among groups (*p* = 0.0022) was observed, with a significant difference between HC and RRMS patients (*p* < 0.001) but not with progressive MS (*p* = 0.51) and between relapsing and progressive MS (*p* = 0.81). Figure 3 reports graphically the longitudinal performance of the three groups of participants on the PASAT-2 at the three assessment points. To conclude, the correlation between HADS-anxiety and SDMT, PASAT-3 and PASAT-2 were low (−0.22, −0.18, −0.12 respectively) and similarly for HADS-depression (−0.23, −0.17, −0.14 respectively). Slight-moderate negative correlations ranging between −0.32 (PASAT-2) and −0.45 (SDMT) were observed with EDSS at baseline.

Lastly, we were still interested in investigating differences in the longitudinal neuropsychological profile between MS patients already cognitively impaired at baseline and MS patients cognitively preserved at the beginning of the study. Considering SDMT, the mean change score over 1 year was 2.51 (standard deviation (SD): 8.3; median:3 [IQR: −2–7]) for 198 no cognitively impaired patients at baseline and with no missing assessment at 1-year while was 1.24 (SD: 10.4; median:1 [IQR: −2–4]) for the 94 cognitively impaired patients at baseline and with no missing observation at 1 year, with no significant difference between the two groups (*p* = 0.064). At the 2-year follow-up, 157 not cognitively impaired patients showed a change from baseline of 0.67 (SD: 7.6; median: 1 [IQR: −5–5]) compared with a delta of −1.2 (SD: 10.4; median: −0.5 [IQR: −5.5–4]) in 72 cognitively impaired patients. The difference did not reach statistical significance (*p* = 0.13). For PASAT-2, at 1 year cognitively and not cognitively impaired patients performed similarly (3 vs. 2; *p* = 0.28) and maintained a similar change also at 2 years (2.5 vs. 3; *p* = 0.63). On PASAT-3, cognitively impaired patients (*n* = 86) improved more (mean: 4.56; SD: 10.5; median:4 [IQR: −1–11]) as compared with no cognitively impaired patients (*n* = 190; mean: 2.47; SD: 8.8; median: 2 [IQR: −2–6]), with a difference that approached the statistical significance (*p* = 0.087). At 2 years however while not cognitively impaired patients maintained a similar change (*n* = 152; mean: 2.3; SD: 10), cognitively impaired patients decreased their values (*n* = 56; mean: 3.4; SD:13.7) with no significant difference between them (*p* = 0.53). Lastly, for WLG significant differences were observed at 1 year: while cognitively impaired patients increased slightly (mean: 1.4; SD: 6), patients without baseline impairments performed better (mean: 3.1; SD: 6.6) with a significant difference between them (*p* = 0.048). No significant differences at 2 years (*p* = 0.90) were detected.

## Discussion

Cognitive deficits typically characterize the neuropsychological profile of patients affected by MS. Robust evidence has shown that a proportion of patients with MS present with some degree of cognitive impairment (Amato et al., 2010; Borghi et al., 2013), urgently calling for the implementation of a neuropsychological assessment as part of routine clinical practice. However, to date studies have not focused on the evolution over time of neuropsychological abilities in large groups of MS patients, as compared to large groups of HC. Besides, at this point in time very little is known about the changes of neuropsychological profile in progressive MS patients, as compared to relapsing-remitting MS patients and HC. In our study, the construct of “cognitive impairment” has been defined operatively as having two or more BRB-N tests with scores at least 1.5 SD below the scores of HC at the corresponding assessment point (e.g., T_0_, T_1_, and T_2_), as widely used in previous studies (Amato et al., 2010; Stankiewicz et al., 2011; Borghi et al., 2013). In keeping with published literature, a significant proportion of MS patients (i.e., 33.9%) presented with some degree of cognitive impairment at the time of recruitment, as compared to HC. At the 1-year follow-up, the proportion of patients with some degree of cognitive impairment increased (39.2%), whereas at the 2-year follow-up it decreased to the baseline value (33.0%). Considering the most critical neuropsychological tests (SDMT, PASAT-2, PASAT-3 and WLG), there was not a significantly different decline over the course of the study between MS patients already compromised at the baseline, and cognitively preserved MS patients (only WLG showed a significant difference). A possible explanation for this is that the time-frame of our study (2 years) be too short to detect a more significant decay of cognitive impairment in patients already impaired at baseline, as compared to patients not presenting with cognitive deficits. Another explanation may be that it is still possible that patients presenting with cognitive impairment at the beginning of the study be more likely to show learning effect than patients not cognitively impaired and then already good at baseline. However, future studies should address this important point.

As we had 29 patients who did not show up at the first follow-up, and an additional 63 patients who did not accept to participate in the second follow-up, a possible explanation of this trend in the proportion of cognitively impaired patients at the three assessment points was that patients more severely affected may have refused to continue the study, allowing us to monitor only patients with a less severe degree of impairment: then, it may be possible that our study has underestimated the decline of cognitive abilities in MS over time. We tested this hypothesis: frequency of cognitively impaired patients who dropped-out at 1-year was 14/109 (12.8%) vs. 14/213 (6.6%) in not cognitively impaired. This difference approached statistical significance (*p* = 0.059). At 2-year follow-up, 36/109 (33%) impaired patients and 55/213 (25.8%) not impaired patients were lost to follow-up, with no statistical significance (*p* = 0.17). Thus, even if the proportion of patients who dropped out was different in the two groups in absolute values, the small proportion of drop-outs which characterized our study did not allow us to detect a statistically significant difference.

The two groups of participants (MS patients and HC) were well matched for pre-morbid IQ, education, and gender. Levels of both anxiety and depression in the two groups were below the clinical borderline range of values, allowing us to rule out on clinical grounds the presence of significant neuropsychiatric symptoms. The two groups still differed in terms of fatigue. Regarding their neuropsychological profile, at baseline patients had significantly lower scores than controls on all of the measures administered, with the exception of the TIB-IQ. At the two scheduled follow-ups, patients got significantly lower scores than controls on all of the measures administered, with the exception of the SRT-CLTR at the 2-year follow-up: this measure detects consistency of the recovery in long-term memory, and it is possible that this specific cognitive ability be less influenced by the progression of MS as compared to others (in fact, this was the only case in which patients got a higher score in absolute value than controls). However, future studies should specifically address this issue more deeply.

The primary aim of our study was to investigate changes in neuropsychological abilities in MS patients over time, as compared to controls. Thus, a LMM was run by considering participants’ performance on each test of the BRB-N at the three assessment points (i.e., T_0_, T_1_ and T_2_) as the within-subjects variable, and “group” (i.e., MS patients and HC) as the between-subjects factor: in all of the measures a significant effect of “group” was detected, as the performance of patients and HC significantly differed in all of the BRB-N tests with patients getting lower scores than controls. However, we were mainly curious about the interaction between “group” and “time”. Interestingly, the interaction between these two factors was statistically significant for the SDMT and the PASAT-2 (that measure mainly attention, working memory, and speed of information processing), and the WLG (that measures semantic verbal fluency), whereas for the PASAT-3 it approached statistical significance. Regarding patients’ performance on the SDMT and PASAT, it is now well established that MS particularly affects the cognitive abilities tapped by these tasks (Chiaravalloti and DeLuca, 2008): our results further extend this consideration, by suggesting not only that these abilities are more vulnerable than others to the presence of MS, but also that they are likely to be more sensitive than others to the progression of the disease, as compared to HC. In keeping with this, in the near future it will be extremely important to focus on pilot studies aiming at training such abilities in MS patients (see for example Hancock et al., 2015), in order to contrast their decay over time.

In addition, we were still interested in studying possible differences in the neuropsychological decline over time of subgroups of patients affected by different course of the disease (relapsing-remitting, RR-MS, vs. progressive course of MS, prog-MS). Thus, for further statistical analyses we considered three groups of participants: HC, patients with a RR-MS, and patients with prog-MS. The interaction between these two factors was statistically significant for the PASAT-3 and the PASAT-2, and the WLG. In keeping with previous literature, patients affected by progressive MS generally got lower scores on neuropsychological tests as compared to patients affected by the relapsing-remitting form of the disease. Regarding the first test (PASAT-3), both the two groups of patients differed significantly from controls, but the comparison of the performance in the two groups of patients did not reach a statistically significant difference. Along a similar vein, regarding the PASAT-2 both the two groups of patients differed significantly from controls, and the comparison of the performance in the two groups of patients approached significance. In our view, the main reason why we were not able to detect a statistically different performance on these tasks in the two groups of patients pertains to the small sample size of the group of patients affected by the progressive form of MS, as compared to the other groups: at the beginning we recruited *N* = 36 prog-MS patients, that remained 29 at the first follow-up, and only 21 at the last scheduled neuropsychological assessment. Thus, even if from a graphical point of view there was a clear tendency of prog-MS patients in getting significantly lower scores than RR-patients in most of the measures over time, it is reasonable to assume that from a statistical standpoint the reduced sample size could have played a role in masking the real entity of such differences.

Lastly, it is still relevant to discuss the pattern of results related to patients’ performance on the WLG task. Verbal fluency deficits can characterize the neuropsychological profile of MS (Vlaar and Wade, 2003; Henry and Beatty, 2006), and it has been recently shown that this cognitive deficit seems to be more frequently associated with the progressive form of the disease (Connick et al., 2012). In keeping with this, in our subgroup of prog-MS patients we were able to demonstrate not only that verbal fluency deficits were present in their neuropsychological profile, but also that this ability appeared to decay significantly over time. However, as previously said, due to the small sample size any conclusion about prog-MS has to be taken with caution, and necessarily need further investigations.

The present study presents some strengths. Firstly, to the best of our knowledge, this is one of the first studies that followed longitudinally large groups of both MS patients and well-matched HC, in order to detect and monitor participants’ neuropsychological profile over time: as a result, the large sample sizes allowed us to obtain reliable evidence about the presence and evolution of cognitive impairment in MS. Previous studies have already included large group of patients (e.g., Van Schependom et al., 2015) or monitored patients over longer period of time (e.g., Piras et al., 2003; Denney et al., 2008), but a strength of our study was the inclusion of large groups of patients and HC, both monitored over 2 years from a neuropsychological point of view. Secondly, only very few patients and HC did not perform all of the tests administered, and above all we were able to maintain into the study the vast majority of participants until the planned 2-year follow-up: thus, we can consider our results as mirroring the entire the samples recruited and not only a small proportion of them. In addition, to minimize practice effects, two parallel versions of the BRB-N were used and were alternatively administered during follow-ups: in doing so, the same version of the neuropsychological battery was repeated by each participant only after 2 years. Lastly, considering not only MS patients as a whole but also splitting the MS patients into two groups according to the type of MS (i.e., RR-MS, and prog-MS) allowed us to investigate the neuropsychological profile associated with the very relevant clinical factor represented by the course of the disease, that should always be taken into account also when dealing with the assessment and rehabilitation of cognitive deficits in MS.

The study also presents some limitations. Firstly, although in the BRB-N the executive functions are underrepresented, due to time constraints we did not administer adjunctive tests of executive functions. Thus, it is not possible to completely rule out the possibility that patients presenting with a mild degree of executive dysfunction may have not been identified as “cognitively impaired” by our neuropsychological assessment, leading to an underestimation of their actual degree of cognitive deficits. Secondly, our final sample of prog-MS patients was relatively small, as compared to RR-MS patients and HC: thus, generalization of conclusions to patients affected by this specific sub-type of disease has to be made with caution. Lastly, we did not investigate the construct of cognitive reserve in our sample, which posits that genetic and environmental factors protect against cognitive decline in the face of neurological disorders, likely due to greater capacity and efficiency of neural circuits. As a result, there are likely to be individual differences in terms of the cognitive changes associated with MS (Stern, 2002).

To conclude, our evidence corroborated on a very large scale that neuropsychological deficits are a common clinical feature of MS that should be carefully screened for at the very early stages of the condition, and above all seriously monitored over time. For the first time it has been shown on a large scale that the cognitive abilities typically compromised at the early stages of MS (attention, working memory, speed of information processing, and verbal fluency) are also more vulnerable than others to the progression of the disease as compared to HC, urgently calling for the identification of effective rehabilitation treatments able to counteract at least partially their decline over time.

## Author Contributions

MB, SC, LO, FS, AB and SM: conceived and designed the experiments. MB, SC, LO, FS and MC: identified the suitable neuropsychological tests. MB, SC and FS: performed the experiments. MC, LP, MP and AS: analyzed the data. MB, SC, FS and MC: contributed reagents/materials/analysis tools. MB, SC, LO, FS, LP, MP, AB, SM, AS and MC: wrote the article. MB, SC, LO, AB and SM: submission to Ethics Committee, collection of medical data, participants’ recruitment.

## Funding

This work was supported by FISM - Fondazione Italiana Sclerosi Multipla (www.aism.it)-Cod. 2009/R/17. The funders had no role in study design, data collection and analysis, decision to publish, or preparation of the manuscript.

## Conflict of Interest Statement

The authors declare that the research was conducted in the absence of any commercial or financial relationships that could be construed as a potential conflict of interest.
